# The impact of job-related stress on township teachers’ professional well-being: A moderated mediation analysis

**DOI:** 10.3389/fpsyg.2022.1000441

**Published:** 2022-10-19

**Authors:** Hongmei Liang, Weichen Wang, Yueyang Sun, Haiying Wang

**Affiliations:** ^1^Faculty of Education, Northeast Normal University, Changchun, China; ^2^School of Psychology, Northeast Normal University, Changchun, China

**Keywords:** township teachers, teachers’ professional well-being, job-related stress, teachers’ professional identity, perceived organizational support

## Abstract

This study aimed to explore the relationship between job-related stress and township teachers’ professional well-being. Based on Job Demand-Resource Model, this study examined the mediating role of teachers’ professional identity and the moderating role of perceived organizational support in this relationship. A total of 24,276 township teachers in China responded to the Teacher Stress Scale, the Teachers’ Professional Identity Scale, the Teachers’ Professional Well-Being Structure Questionnaire, and the Perceived Organizational Support Scale. Results showed that: (1) the professional well-being of township teachers differed significantly in terms of school type and demographic characteristics like age and gender; (2) job-related stress negatively predicted township teachers’ professional well-being, with teachers’ professional identity playing a mediating role; (3) the relation between job-related stress and teachers’ professional identity was moderated by perceived organizational support; and (4) in the moderated mediation analysis, job-related stress positively predicted township teachers’ professional well-being. These findings indicated that township teachers’ professional well-being was influenced by both organizational and individual factors, which provided a theoretical basis and intervention pathways for improving township teachers’ professional well-being.

## Introduction

Well-being, particularly a teacher’s professional well-being, is a positive emotional state and a harmonious relationship between specific contexts and the individual (Van Petegem et al., 2005). In the PISA 2021 framework, the analysis of teachers’ professional well-being published by the Organization for Economic Co-operation and Development (OECD) introduced teachers’ professional well-being as their responses to certain aspects related to their work and profession, which are evaluated based on four dimensions: cognitive well-being, subjective well-being, physical and mental health well-being, and social well-being (Viac and Fraser, 2020). The professional well-being of school teachers impact the stability and the development of the teacher workforce. Further, a growing number of studies have discovered that professional well-being not only affects teachers’ teaching performance but also affects students’ learning motivation (Hiver and Dörnyei, 2015), personality formation, academic achievement, and ability development (Turner and Thielking, 2019; Penttinen et al., 2020; Hu and Wang, 2022). Among other things, job-related stress has been widely concerned as a key risk factor affecting teachers’ professional well-being, as teachers often experience more significant (job-related stress than partitioners in other professions (Wang et al., 2014; Darmody and Smyth, 2016). Compared with urban areas, the relatively poor living and working conditions have heavily increased township teachers’ job-related stress and weakened their group stability. This study focused on township teachers’ professional well-being and explored the indirect pathways through in which job-related stress affects them in order to provide a theoretical basis and intervention approach for improving the professional well-being of township teachers.

Job-related stress is an individual’s physiological, psychological, and behavioral response to a work environment, and it reflects an imbalance in the relationship between the individual and their working environment that leads to an unpleasant emotional experience at work (McGrath, 1976; Lazarus and Folkman, 1984). According to the Job Demands-Resources Model (JD-R) (Demerouti et al., 2001), the organizational, social, physical, and psychological demands that employees encounter in the workplace can lead to burnout by negatively impacting the person’s physical and mental health. In the school setting, teachers’ psychological well-being can be negatively affected by work demands such as workload, time constraints, and the psychological environment (Ibrahim et al., 2021). Poormahmood et al. (2017) found that the more stressful the work of primary school teachers, the lower the well-being engage in. Most prior studies in the Chinese social-cultural context have shown that job-related stress was negatively related to teachers’ professional well-being. The results of studies comprising diverse regional groups of primary and secondary school teachers showed that job-related stress negatively predicted their professional well-being (Yao et al., 2016; Li, 2020; Zhu et al., 2022). Also, existing studies on the early childhood teacher found that job-related stress had an negatively effect on professional well-being (Wang et al., 2014; Li and Zhang, 2019; Liu et al., 2022). Contrary to these findings, Dai (2020) observed that job-related stress negatively predicted teachers’ professional well-being directly, but it also exhibited a positive predictive effect on teachers’ professional well-being through psychological empowerment. Boshoff et al. (2014) et al. studied Black South Africa teachers and showed that job-related stress positively predicted teachers’ professional well-being. As the participants were recruited in China, this study based on previous studies in Chinese context and the JD-R model proposed hypothesis1: Job-related stress is negatively related to township teachers’ professional well-being and has a significant negative predictive effect.

As the relationship between job-related stress and professional well-being has been studied, some researchers have suggested that certain factors, such as professional identity, may buffer the impact of job-related stress on teachers’ professional well-being (Wang et al., 2014). The teachers’ professional identity is characterized as a teacher’s positive attitudes related to the profession, a teacher’s perception of “who we are” or “what we think we are,” and a process of identity formation for the teacher’s own role and its development (Smagorinsky et al., 2004). The Job Demands–Resources Model (Demerouti et al., 2001) suggests that job resources can alleviate individual burnout. Professional identity is an essential internal individual resource for teachers, which constitutes a critical component that positively influences teachers’ professional well-being (Qiao, 2012; Wang et al., 2018; Su, 2021; Wei et al., 2021; Liu et al., 2022). Moreover, teachers’ professional identity also has indirect effects on their professional well-being through mediating variables such as teachers’ work engagement and social support (Li et al., 2021). In a study of 267 teachers in western China, professional identity was found to influence teacher burnout through work satisfaction (Lu et al., 2022), which is a significant negative indicator of their well-being (Yang et al., 2015). Teachers’ professional identity develops as a result of the interactions between teachers and others in their work environment and is subject to the influence of their external surroundings (Wei, 2008). Increased job-related stress may cause a reduction in teachers’ professional identity (Peng, 2021). Heavy workload, poor living conditions, low benefits, low social status, and few promotion opportunities can all elevate township teachers’ job-related stress levels. Excessive stress weakens teachers’ professional identity, with negative coping strategies partially mediating the relationship between job-related stress and teachers’ professional identity (Wang and Zhang, 2017) and resilience acting as a moderator (Zhou and Ning, 2020). Additional tasks in schoolwork increase teachers’ workload and generate more job-related stress, which affects their professional identity and diminishes their professional well-being (Akyurek and Can, 2022). Subsequently, the decline in teachers’ professional identity affects the professional well-being of township teachers by reducing their positive experiences and behaviors, which leads to burnout and a tendency to resign (Akyurek and Can, 2022; Hamelin et al., 2022). Therefore, this study proposes hypothesis 2: Teachers’ professional identity mediates the relationship between job-related stress and township teachers’ professional well-being.

According to the Conservation of Resource Theory, the organizational support for workers has essential core values and may significantly influence how they perform at work and behave in general (Hobfoll, 2001, 2011). An individual’s perceived organizational support measures how much the organization values their contribution to the organization and how much the organization cares about their well-being (Ling et al., 2006; Wang et al., 2020a). Teachers’ perceived organizational support has a noticeable impact on their professional identity formation (Lam et al., 2016). Specifically, the perceived organizational support relates to the administration and management, code of conduct, developmental guidelines, resource assistance, and humanistic care that the teachers experience in the school organization (Wang et al., 2020a). Teachers with high perceived organizational support not only experience caring and recognition from the organization but also possess higher job engagement (Carrell et al., 2022) and professional recognition (Su, 2021). Hence, perceived organizational support might moderate the effects of job-related stress on teachers’ professional identity. Increased job-related stress can result in undesirable outcomes such as emotional dysregulation in individuals. However, employees’ perceived organizational support can bolster the sense of self-value, thus minimizing the negative stress caused by the overwhelming job demands (Allen, 2001; Karatepe, 2011; Harris et al., 2013). The Job Demands-Resources Model proposes that sufficient work resources can relieve burnout and positively anticipate employees’ professional well-being (Demerouti et al., 2001), and they can also effectively counteract the negative impacts of job demands (Bakker and Demerouti, 2007). External work resources, including disciplinary commitment (Agago, 1995), family support (Nasurdin and O'Driscoll, 2012), organizational systems (Harris et al., 2013), and socioeconomic factors (Nazari and Oghyanous, 2021), are also effective in moderating the effects of job-related stress on professional well-being. As an essential dimension of work resources, perceived organizational support may moderate the relationship between job-related stress and township teachers’ professional well-being. A heightened perception of organizational support enhances teachers’ identity and commitment to their organization, strengthens their willingness to contribute, and thus improves teachers’ psychological well-being (Kurtessis et al., 2017). The professional well-being of special education teachers is directly and positively predicted by perceived organizational support and indirectly influenced by psychological capital, emotional exhaustion, etc. (Wang et al., 2020a,b). Therefore, perceived organizational support is an important external factor affecting variables including psychological capital, emotional exhaustion, and job satisfaction of township teachers. In addition to moderating the effect of job-related stress on township teachers’ professional well-being, the perceived organizational support may also moderate the impact of job-related stress on teachers’ professional identity and thus further affect professional well-being. Therefore, our study postulates hypothesis 3: Perceived organizational support moderates the direct path and the first half of the mediating process of job-related stress → teachers’ professional identity → township teachers’ professional well-being.

In summary, based on the Job Demands-Resources Model and Resource Conservation Theory, this study conducted an in-depth investigation of the relationship between job-related stress and township teachers’ professional well-being through a moderated mediation analysis (Supplementary Figure 1). The main research aims of the present paper include the following three: (1) to explore whether job-related stress negatively predicts township teachers’ professional well-being; (2) to investigate whether professional identity can play a mediating role between job-related stress and professional well-being; 3) to examine whether perceived organizational support moderates the relationship between job-related stress and township teachers’ professional well-being and the relationship between job-related stress and teachers’ professional identity. These hypotheses can be examined to explore further the relationship between job-related stress and township teachers’ professional well-being, establish a referenceable theoretical framework for enhancing township teachers’ professional well-being, and provide effective interventions for certain influencing factors.

## Materials and methods

### Participants

This study was conducted with township primary and secondary school teachers as the research subjects, and the survey sample covered 31 provinces and cities across China. The survey was carried out in the form of an electronic questionnaire. The subjects were required to complete questionnaires as in-service township teachers and were screened according to their employment schools. A total of 24,823 questionnaires were collected from in-service township primary and secondary school teachers. The subjects took about 30 min to fill out the questionnaires. After eliminating 547 invalid samples according to the minimum response time, the final number of valid questionnaires was 24,276, with an efficiency rate of 97.80%. The final valid subjects included 18,085 female teachers (74.5%) and 6,191 male teachers (25.5%). Details of the study subjects are shown in Supplementary Table 1. The study was approved by the Academic Ethics Committee of the College of Psychology of Northeast Normal University.

### Measures

#### Job-related stress

The Teacher Stress Scale developed by Boyle et al. (1995) was used to measure township teachers’ job-related stress. The scale consists of two dimensions: workload and student misconduct. The workload dimension has 2 items, such as “too much work to do (e.g., lesson preparation and marking)” and student misconduct consists of 5 items, such as “managing classroom discipline.” Participants rated the items on a 5-point Likert-type scale (1 = strongly inconsistent; 5 = strongly consistent), with a higher total score indicating that teachers perceived higher stress. The Cronbach’s alpha was 0.714.

#### Teachers’ professional well-being

The Structure Questionnaire of Teachers’ Professional Well-being developed by Li and Gai (2022) was adopted to measure township teachers’ professional well-being. The scale consists of 65 items categorized into four dimensions: subjective well-being (19 items, e.g., “You feel happy that many things you did have a positive impact on others”), cognitive well-being (20 items, e.g., “You have the flexibility to evaluate student development in a variety of ways”), physical well-being (9 items, e.g., “You have frequent headaches or stomach pains”), and social well-being (17 items, e.g., “You feel that all sectors of society respect teachers”). The 5-point Likert-type scale (1 = strongly inconsistent; 5 = strongly consistent) was adopted, and 14 items in the questionnaire were scored reversely. The higher the total scores are, the higher the teachers’ professional well-being. The Cronbach’s alpha was 0.974.

#### Teachers’ professional identify

The Teachers’ Professional Identify Scale compiled by Wei et al. (2013) was used to assess teachers’ professional identity. The scale consists of 18 items and is divided into four dimensions. Three of these dimensions were selected for measurement according to the purpose of the study, which are professional value, role value, and sense of belonging. The screened 12 items were rated on a 5-point Likert-type scale (1 = strongly inconsistent; 5 = strongly consistent). The higher scores indicate a high level of professional identity. The Cronbach’s alpha was 0.866.

#### Perceived organizational support

The Perceived Organizational Support Scale of Employees developed by Ling et al. (2006) was used to evaluate township teachers’ organizational support from two dimensions: working support and concern for benefits. The survey questions were modified to accommodate the characteristics of the participant group. Through exploratory factor analysis, the scale delates 2 items that the factors were less than 0.4 after principal component analysis and finally consisted of 15 items. Participants rate items on a 5-point Likert-type scale (1 = strongly inconsistent; 5 = strongly consistent), with higher ratings indicating a higher level of perceived organizational support for teachers. The Cronbach’s alpha was 0.927.

### Data analysis

The researchers distributed the questionnaires online, and the teachers in the township answered the questionnaires voluntarily. The SPSS25.0 software and the SPSS PROCESS macro program were used for data processing. Descriptive statistics, variance analysis, and Pearson correlation were performed to examine the differences in professional well-being scores with distinctive characteristics. Based on 5,000 bootstrap samples, the mediation effect of professional identity between job-related stress and teachers’ professional well-being was analyzed using the PROCESS macro (Model 4) developed by Hayes (2013). Finally, we used the PROCESS macro (Model 8) to examine whether perceived organizational support moderated this mediation process. The effects are significant when the confidence intervals exclude zero.

## Results

Due to objective conditions, only self-reported methods were utilized to collect data information in this study, which may address an issue of common method variation (CMV). To minimize the possible bias, procedural control methods, including anonymous measurement and reversed questions on selected items, were applied according to suggestions Zhou and Long (2004). In the data processing, Harman’s single-factor test for CMV was employed. Each item of the four scales was separately subjected to exploratory factor analysis (EFA), and 12 factors with eigenvalues greater than 1 were extracted. The cumulative variance explained by the first factor was 38.54%, which was less than the critical value of 40%, indicating that there was no significant CMV (Zhou and Long, 2004).

### Variance analysis of township teachers’ professional well-being

#### Demographic characteristics in relation to teachers’ professional well-being

There were significant differences in the professional well-being of township teachers in terms of demographic variables, including gender, age, educational background, working experience, professional title, and teaching period. Specifically, there was a significant gender difference in the professional well-being of township teachers (*t* = −3.405, *p* < 0.001), and the well-being of male teachers (*M* = 238.915, *SD* = 39.451) was significantly lower than that of female teachers (*M* = 240.823, *SD* = 37.582).

The results of variance analysis showed that township teachers’ professional well-being was a significant difference in terms of age (*F*_(7,24,268)_ = 90.928, *η*^2^ = 0.026, *p* < 0.001), educational background (*F*_(2,24,273)_ = 125.082, *η*^2^ = 0.010, *p* < 0.001), working experience (*F*_(8,24,267)_ = 100.892, *η*^2^ = 0.032, *p* < 0.001), professional title (*F*_(4,24,271)_ = 130.277, *η*^2^ = 0.021, *p* < 0.001) and teaching period (*F*_(2,24,273)_ = 173.955, *η*^2^ = 0.014, *p* < 0.001).

Township teachers aged 56–60 have the highest professional well-being (*M* = 258.569, *SD* = 37.020), significantly higher than teachers of other ages. Township teachers aged 36–40 had the lowest professional well-being (*M* = 234.832, *SD* = 37.524), significantly lower than those aged 21–25 (*M* = 238.880, *SD* = 38.759), 41–45 (*M* = 239.295, *SD* = 37.068), 46–50 years old (*M* = 242.449, *SD* = 37.808), 50 ~ 55 years old (*M* = 249.761, *SD* = 38.438) and 56–60 years old teachers.

Township teachers with an associate degree or below had the highest professional well-being (*M* = 246.695, *SD* = 39.152). In contrast, those with master’s and doctoral degrees had the lowest professional well-being (*M* = 235.417, *SD* = 38.075), and the difference was significant. The professional well-being of teachers with a bachelor’s degree (*M* = 238.105, *SD* = 37.486) is significantly lower than that of teachers with an associate degree or below but also not significantly higher than that of teachers with master’s and doctoral degrees.

Township teachers with 36–40 years of teaching experience had higher professional well-being (*M* = 259.429, *SD* = 37.367) than others. Teachers with 3 to 5 years of experience reported the lowest professional well-being (*M* = 232.683, *SD* = 37.177), significantly lower than other teachers except those with 11 to 15 years of experience (*M* = 233.563, *SD* = 36.817).

Regarding the professional title, senior teachers experienced the highest professional well-being (*M* = 249.227, *SD* = 37.643), significantly higher than teachers holding lower-ranking positions. Level II teachers had the lowest professional well-being (*M* = 233.198, *SD* = 36.698), which is considerably lower than that of the ungraded (*M* = 245.121, *SD* = 38.281), Level I (*M* = 239.952, *SD* = 38.048) and senior-titled teachers, but not significantly different from the Level III teachers (*M* = 234.828, *SD* = 38.946).

In terms of the teaching period, the highest level of professional well-being was found among teachers in primary schools (*M* = 243.011, *SD* = 38.408), which is significantly higher than that of junior high school teachers (*M* = 232.779, *SD* = 35.843) and senior high school teachers (*M* = 232.392, *SD* = 36.558). The results of junior high school teachers and senior high school teachers do not significantly differ from one another.

#### School characteristics in relation to teachers’ professional well-being

In this study, the schools in which the teachers worked were categorized by two factors: (1) public schools vs. private schools, and (2) day schools vs. boarding schools. The findings of the different analyses showed that township teachers’ professional well-being varied significantly depending on these variables. Results from the independent samples t-test showed a significant difference in township teachers’ professional well-being regarding school type. The teachers in public school reported significantly higher professional well-being (*M* = 240.417, *SD* = 38.103) than teachers in private schools (*M* = 236.056, *SD* = 36.341). Township teachers’ professional well-being also significantly differed on whether the school was a boarding school (*t* = −7.473, *p* < 0.001). The professional well-being of teachers in boarding schools (*M* = 238.018, *SD* = 37.679) was significantly lower than that of teachers in non-boarding schools (*M* = 241.772, *SD* = 38.250).

### Descriptive statistics and correlation analysis

As shown in Table 1, job-related stress was found to be negatively correlated with perceived organizational support, teachers’ professional identity, and township teachers’ professional well-being (*p* < 0.001). Teachers’ professional identity was found to be positively correlated with perceived organizational support (*p* < 0.001) and township teachers’ professional well-being (*p* < 0.001). Perceived organizational support was also positively associated with township teachers’ professional well-being (*p* < 0.001). To avoid the influence of demographic variables and other factors such as the characteristics of the school on the study results, they were analyzed as control variables in the subsequent analysis.

**Table 1:** Means, standard deviations, and correlations among variables.

	*M*	*SD*	1	2	3	4
JS	16.042	4.035	1			
POS	54.321	10.799	−0.643^***^	1		
TPI	46.217	6.639	−0.686^***^	0.671^***^	1	
TTPWB	240.337	38.075	−0.537^***^	0.778^***^	0.734^***^	1

(1) *N* = 24,276; (2) ***p < 0.001; (3) JS, job-related stress; POS, perceived organizational support; TPI, teachers’ professional identity; TTPWB, township teachers’ professional well-being.

### The mediating role of teachers’ professional identity

To test hypothesis 2, the mediating effect of teachers’ professional identity between job-related stress and professional well-being was examined using the PROCESS model 4. The outcomes of the regression tests are summarized in Table 2. Job-related stress was found to have a significant negative effect on teachers’ professional well-being (*β* = −0.526, *p* < 0.001, 95% CI = [−0.5362, −0.5153]). After professional identity was incorporated as a mediating variable into this equation, the negative predictive effect of job-related stress on teachers’ professional well-being (*β* = −0.059, *p* < 0.001, 95% CI = [−0.0720, −0.0449]) and professional identity (*β* = −0.683, *p* < 0.001, 95% CI = [−0.6952,−0.6709]) were both still significant, and teachers’ professional identity positively predicted professional well-being (*β* = 0.684, *p* < 0.001, 95% CI = [0.6711, 0.6971]). Thus, teachers’ professional identity was found to play a partial mediating role between job-related stress and teachers’ professional well-being (Supplementary Figure 2).

**Table 2:** Testing the mediation effect of teachers’ professional identity between job-related stress and teachers’ professional well-being.

	TTPWB				TPI				TTPWB			
	*β*	SE	*t*	95% CI	*β*	SE	*t*	95% CI	*β*	SE	*t*	95% CI
Gender	0.114	0.013	8.761^***^	[0.0885,0.1396]	0.095	0.012	8.277^***^	[0.0726,0.1176]	0.049	0.011	4.621^***^	[0.0282,0.0698]
Age	0.104	0.009	12.146^***^	[0.0872,0.1208]	0.040	0.008	5.170^***^	[0.0248,0.0552]	0.077	0.007	11.295^***^	[0.0633,0.0899]
Working Experience	−0.027	0.008	−3.576^***^	[−0.0416,-0.0122]	−0.016	0.007	−2.312^*^	[−0.0293,-0.0024]	−0.016	0.006	−2.691^**^	[−0.0278,-0.0044]
Educational Background	−0.045	0.013	−3.310^***^	[−0.0709,-0.0182]	0.012	0.012	1.028	[−0.0107,0.0344]	−0.053	0.011	−4.886^***^	[−0.0737,-0.0315]
Professional Title	−0.078	0.007	−10.652^***^	[−0.0921,-0.0635]	−0.016	0.006	−2.560^*^	[−0.0289,-0.0038]	−0.067	0.006	−11.320^***^	[−0.0782,-0.0551]
Teaching Period	0.107	0.010	10.737^***^	[0.0876,0.1267]	0.043	0.009	4.758^***^	[0.0252,0.0606]	0.078	0.008	9.535^***^	[0.0618,0.0938]
Boarding School	0.003	0.012	0.221	[−0.0209,0.0262]	−0.033	0.010	−3.194^**^	[−0.0538,-0.0129]	0.026	0.010	2.621^**^	[0.0064,0.0445]
Types of School	−0.011	0.038	−0.282	[−0.0846,0.0633]	−0.008	0.034	−0.241	[−0.0751,0.0586]	−0.005	0.031	−0.160	[−0.0664,0.0564]
JS	−0.526	0.005	−98.403^***^	[−0.5362,-0.5153]	−0.683	0.006	−110.071^***^	[−0.6952,-0.6709]	−0.059	0.007	−8.467^***^	[−0.0720,-0.0449]
TPI									0.684	0.007	103.410^***^	[0.6711,0.6971]
*R* ^2^	0.308				0.473				0.554			
*F*	1275.007^***^				1429.595^***^				2736.531^***^			

(1) *N* = 24,276; (2) ***p < 0.001. **p < 0.01; *p<0.05; (3) JS, job-related stress; POS, perceived organizational support; TPI, teachers’ professional identity; TTPWB, township teachers’ professional well-being.

### The moderating effect of perceived organizational support

To examine hypothesis 3, the study first investigated whether the relationship between job-related stress and teachers’ professional identity was moderated by perceived organizational support. The results in Table 3 demonstrated that job-related stress has a significant negative predictive effect on teachers’ professional identity (*β* = −0.441, *p* < 0.001) and a significant positive predictive effect on township teachers’ professional well-being (*β* = 0.140, *p* < 0.001). The interaction of job-related stress with perceived organizational support did not show a significant effect on teachers’ professional well-being (*β* = 0.003, *p* > 0.05), but significantly and adversely predicted teachers’ professional identity (*β* = −0.057, *p* < 0.001). This suggested that perceived organizational support moderated the relationship between job-related stress and teachers’ professional identity.

**Table 3:** Testing the moderated mediation effect of perceived organizational support.

	TPI			TTPWB		
	*β*	SE	*t*	*β*	SE	*t*
Gender	0.085	0.010	8.201^***^	0.049	0.009	5.706^***^
Age	0.026	0.007	3.792^***^	0.067	0.006	12.153^***^
Working Experience	−0.001	0.006	−0.140	0.002	0.005	0.369
Educational Background	0.020	0.010	1.938	−0.036	0.009	−4.143^***^
Professional Title	−0.010	0.006	−1.775	−0.060	0.005	−12.500^***^
Teaching Period	−0.017	0.008	−2.068^*^	0.004	0.007	0.612
Boarding School	−0.033	0.009	−3.522^***^	0.018	0.008	2.227^*^
Types of School	−0.013	0.032	−0.406	−0.012	0.026	−0.472
JS	−0.441	0.007	−64.184^***^	0.140	0.006	23.258^***^
TPI				0.440	0.006	70.511^***^
POS	0.405	0.007	60.759^***^	0.565	0.006	92.849^***^
JS × POS	−0.057	0.006	−9.016^***^	0.003	0.003	0.815
*R* ^2^	0.569			0.707		
*F*	2366.478^***^			4227.991^***^		

(1) *N* = 24,276; (2) ***p < 0.001. *p < 0.05; (3) JS, job-related stress; POS, perceived organizational support; TPI, teachers’ professional identity; TTPWB, township teachers’ professional well-being.

Then, this study further examined the mechanism of the moderating effect of perceived organizational support in the relation between job-related stress and teachers’ professional identity. A simple slope of the moderating effect was plotted, with one *SD* above the mean score for the high group and one *SD* below the mean score for the low group, as presented in Supplementary Figure 3. For the township teachers with low perceived organizational support, teachers’ professional identity showed a significant downward trend with an increase in job-related stress (*β*_high perceived organizational support_ = −0.498, *t* = −9.083, *p* < 0.001). However, for the township teachers with high perceived organizational support, teachers’ professional identity showed a more prominent downward trend as job-related stress increased (*β*_low perceived organizational support_ = −0.384, *t* = −9.083, *p* < 0.001).

## Discussion

Previous studies have shown that township teachers’ professional well-being is critical to both the stability of the teaching workforce and the development of primary and secondary school students. Based on the Job Demands-Resource Model, this study analyzed the relationship between job-related stress and the professional well-being of township teachers at both the organizational and individual levels.

### Demographic variance analysis

The results of the variance analysis have suggested that township teachers’ professional well-being was significantly different in terms of gender, age, educational background, working experience, professional title, and teaching period of township teachers, and the characteristics of schools.

Firstly, male township teachers showed significantly lower professional well-being than female township teachers. Influenced by traditional Chinese cultural concepts, men had been supposed to provide most of the household economic support, earn higher wages and enjoy a higher social status, while women had been primarily in charge of tasks like childrearing and teaching (Liu, 2011). The peculiarities of the working environment and profession result in township teachers’ low income and inferior social status, which deviates from the traditional view of men’s roles. As a result, male township teachers reported a lower sense of professional well-being.

Secondly, teachers in the 56–60 age range reported the highest professional well-being, and teachers in the 36–40 age range reported the lowest. On the one hand, middle-aged township teachers aged 36–40 usually face problems related to job advancement and skill improvement, leading them to suffer more pressure from career development. On the other hand, township teachers aged 36–40 may also experience more family-related stress. Since the teaching experience and professional titles are strongly related to the age of township teachers, those with 36–40 years of teaching experience and those with senior titles have the highest professional well-being (Xie et al., 2019).

Thirdly, the professional well-being of township teachers with master’s degrees appeared to be the lowest, whereas those with college degrees and below reported the highest professional well-being. For township teachers with graduate degrees, the disparity between their educational level and socioeconomic status leads to the low job satisfaction and job fulfillment of the highly educated teachers, which in turn affects their professional well-being.

In addition, primary school teachers in the township reported the highest professional well-being. With fewer instructional tasks and administrative work, primary school teachers bear less academic pressure, enjoy more leisure time, and thus have the highest professional well-being. Public school teachers showed higher professional well-being than private school teachers. This is mainly because teaching in public schools is less stressful since public school teachers are recruited as institutional personnel and thus have more stable employment than teachers in public school. Similarly, boarding school township teachers are not only responsible for the academic performance of their students but also for managing students’ accommodation and living arrangements within the campus. The workload of boarding school teachers is more extensive and stressful. Consequently, their professional well-being is greater than non-boarding school township teachers.

### The relationship between job-related stress and teachers’ professional well-being

This study found that job-related stress was significantly and negatively related to professional well-being. In the mediation model, job-related stress negatively predicted township teachers’ professional well-being. The results supported hypothesis 1, which is consistent with prior research (Poormahmood et al., 2017; Ibrahim et al., 2021). In recent years, the government has increased attention and investment in township education, which has raised the social status of teachers and markedly improved their working environment, compensation, and living conditions, but township teachers continue to confront extracurricular tasks and various career development challenges (Liao et al., 2021). As education reform continues to progress, the demands on the teaching profession are becoming more diverse and elaborate. Besides assuring the quality of teaching and students’ academic achievement, teachers are also expected to pay attention to students’ mental health conditions. Due to the particularity of townships, with a high percentage of children experiencing left-behind experiences, teachers in townships have to pay more attention to students’ everyday life and psychological needs in addition to their regular work. As a result, township teachers are under long-term job-related pressure and thus more likely to have a negative view of their professional well-being (Yao et al., 2016; Zhu et al., 2022).

However, after adding the moderating effect of perceived organizational support, job-related stress positively predicted the professional well-being of township teachers, which is consistent with the result of prior research (Dai, 2020). Boshoff et al. (2014) indicated that a group of black teachers in South Africa still reported high levels of professional well-being while experiencing high levels of job-related stress. This outcome may be impacted by a combination of sociocultural context and intra-individual variables. Individuals’ perceptions of job-related stress are affected by race and sociocultural context (Jackson et al., 2010). Individuals from collectivist versus individualist contexts define the relationship between themselves and the environment differently, which are related to their own traits such as self-concept, cognitive style, etc. (Oyserman and Lee, 2008). Individuals with higher levels of stable traits such as positive coping styles and psychological capital are more prone to perceive the stress as manageable and even motivating (Wang and Zhang, 2017; Dai, 2020; Wang et al., 2020a), i.e., eustress (Nelson and Simmons, 2003). Positive job-related stress may contribute to higher professional well-being, good physical and mental condition, and efficient performance. For example, Lee et al. (2019) found that job-related stress can promote work motivation, and that positive work motivation can increase professional well-being. Chinese township teachers are more committed to collectivism and emphasize contributing to the common good. As a result, people are expected to act in ways consistent with the community’s social demands and expectations. Therefore, in the context of collectivism, the perceived job-related stress is more likely to interact with an individual’s internal stability factors as the external environment changes. This interaction may positively affect township teachers’ professional well-being, especially when the environment changes are closely related to their profession.

### The mediating effect of professional identity

According to the results, teachers’ professional identity also served as a partial mediator in the relationship between job-related stress and township teachers’ professional well-being. This supported hypothesis 2 and is consistent with those of earlier studies (Wang et al., 2014). Job-related stress is the perception of stress in the external work environment (Lazarus and Folkman, 1984). A certain amount of stress can be beneficial for maintaining and enhancing individual psychological health and improving productivity at work, but in some instances, excessive stress can result in various psychological symptoms or disorders (Ganster and Rosen, 2013). Primary and secondary school teachers are frequently supposed to take on extra administrative work, especially in townships, which significantly increases the workload and raises job-related stress. When teachers are in a long-term stressful work scenario and unable to manage it, detrimental influences may occur (Chen and Xu, 2000) and, in turn, reduce their professional identity (Zhou and Ning, 2020; Cheng, 2021; Peng, 2021). The Job Demand-Resource Model stated that job resources, including feedback, rewards, and job control, could markedly alleviate job burnout. Teachers’ professional identity, defined as a positive career-related attitude of teachers, is an essential individual resource that reinforces teacher engagement, improves teachers’ performance (Li et al., 2021), and thus increases township teachers’ professional well-being. Job satisfaction also has an indirect impact on this process (Lu et al., 2022). In conclusion, enhancing township teachers’ professional identities can stimulate positive behaviors and help them cope effectively with job-related stress, which can elevate their professional well-being (Akyurek and Can, 2022; Hamelin et al., 2022).

### The moderating effect of perceived organizational support

This study also found that perceived organizational support plays an important role in moderating the indirect effects of job-related stress on professional well-being. The results showed that perceived organizational support moderated the relationship between job-related stress and teachers’ professional identity but did not have a significant moderating effect on the relationship between job-related stress and township teachers’ professional well-being, which partially supported hypothesis 3. Perceived organizational support is an important external work resource in forming professional identity (Hobfoll, 2001, 2011; Lam et al., 2016) and can reduce the adverse effects of job-related stress (Allen, 2001; Karatepe, 2011; Harris et al., 2013). In this study, perceived organizational support mitigated the adverse effects of job-related stress on teachers’ professional identity, whereby township teachers with high perceived organizational support exhibited significantly higher professional identity than those with low perceived organizational support in the presence of high job-related stress. Additionally, the moderating effect of perceived organizational support on the direct link between job-related stress and township teachers’ professional well-being was insignificant. The explanation for this might be that township teachers’ perceived organizational support is the measure of whether the organization appreciates their contributions and how much the organization cares about them (Ling et al., 2006), which is more strongly associated with their professional identity than their professional well-being (Wang et al., 2020a). Thus, perceived organizational support is more likely to indirectly improve township teachers’ professional well-being by alleviating negative cognition associated with job-related stress.

## Conclusion

Based on the Job Demand-Resource Model, this study explored the relationship between job-related stress and township teachers’ professional well-being. The results showed that teachers’ professional identity mediated the relationship between the two, and job-related stress negatively predicted township teachers’ professional well-being. In the moderated mediation model, job-related stress positively predicted teachers’ professional well-being, with perceived organizational support moderated the link between job-related stress and professional identity. Even though this study has contributed to illustrating how job-related stress affects teachers’ professional well-being in Chinese townships, there are still a few deficiencies in the study. First, the study utilized an existing questionnaire to evaluate teachers’ job-related stress, which was not comprehensive enough for identifying job-related stress sources. Therefore, future researchers could adopt qualitative research to determine the primary sources of the job-related stress that significantly impact township teachers and thus more precisely evaluate the level of job-related stress experienced by township teachers. Second, owing to spatial and temporal limits, this study adopted a cross-sectional research methodology, and as a result, there are still constraints for explaining the causality. The outcomes of this study can be expanded upon and further validated in the future using a longitudinal method. Finally, this study only analyzed the effect of teachers’ professional identity among individual factors. Future studies could examine the interactions of internal stability factors and external factors, such as psychological resilience and teachers’ coping styles with the work environment, and further explain the positive effects of job-related stress on the prediction of township teachers’ professional well-being.

This study’s findings can provide suggestions for enhancing the professional well-being of township teachers. First, schools and governmental institutions should clarify teachers’ responsibilities as educators in less developed regions and instruct them to manage job-related stress positively. Schools should also dedicate themselves to establishing a harmonious campus to minimize teachers’ burdens from various perspectives, enhance township teachers’ professional identity, and increase their professional well-being. Second, organizations should increase the level of support for teaching staff, improve their benefits and treatment, appreciate their work achievements, and acknowledge their value to society. When teachers’ perceived organizational support improves, they experience more respect, understanding, and positive expectations from the school and community, which leads to a higher professional identity and eventually becomes a valuable resource for relieving job-related stress and improving their professional well-being.

## Data availability statement

The raw data supporting the conclusions of this article will be made available by the authors, without undue reservation.

## Ethics statement

The studies involving human participants were reviewed and approved by the Academic Ethics Committee of the College of Psychology of Northeast Normal University. The participants provided their electronic informed consent to participate in this study.

## Author contributions

HL designed the research. WW wrote the manuscript and analyzed the data. YS modified the manuscript. HL and HW recruited the participants. All authors contributed to the article and approved the submitted version.

## Conflict of interest

The authors declare that the research was conducted in the absence of any commercial or financial relationships that could be construed as a potential conflict of interest.

## Publisher’s note

All claims expressed in this article are solely those of the authors and do not necessarily represent those of their affiliated organizations, or those of the publisher, the editors and the reviewers. Any product that may be evaluated in this article, or claim that may be made by its manufacturer, is not guaranteed or endorsed by the publisher.
