# Supplementary material for: The impact of job-related stress on township teachers’ professional well-being: A moderated mediation analysis
Source: Front Psychol. 2022 Oct 19;13:1000441. doi: 10.3389/fpsyg.2022.1000441 (PMC9629836; doi:10.3389/fpsyg.2022.1000441)
Supplement: Supplementary file 1 [file Table_1.DOCX]

Supplementary Material

# Supplementary Figures and Tables

## Supplementary Figures


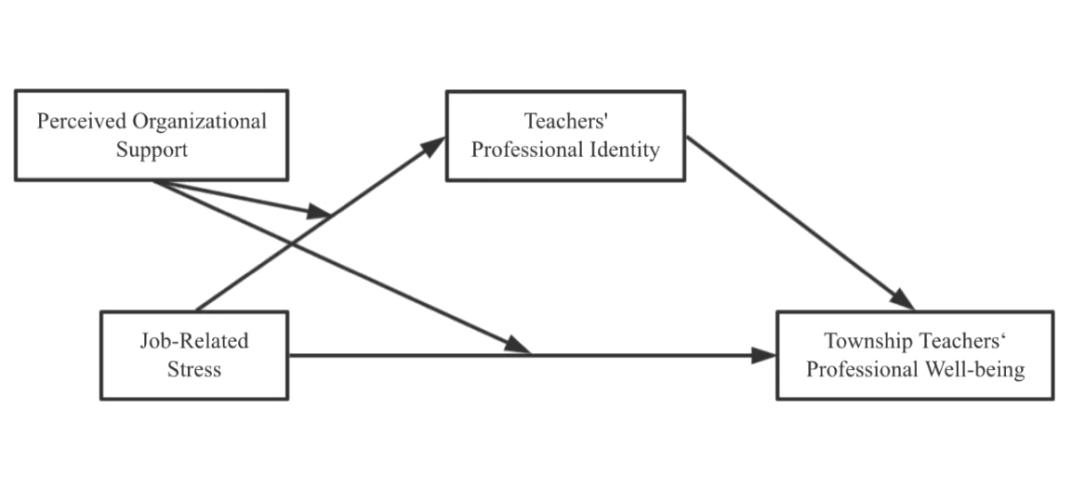


**Supplementary Figure 1.** The moderated mediation effect among job-related stress, township teachers’ professional well-being, teachers’ professional identity, and perceived organizational support


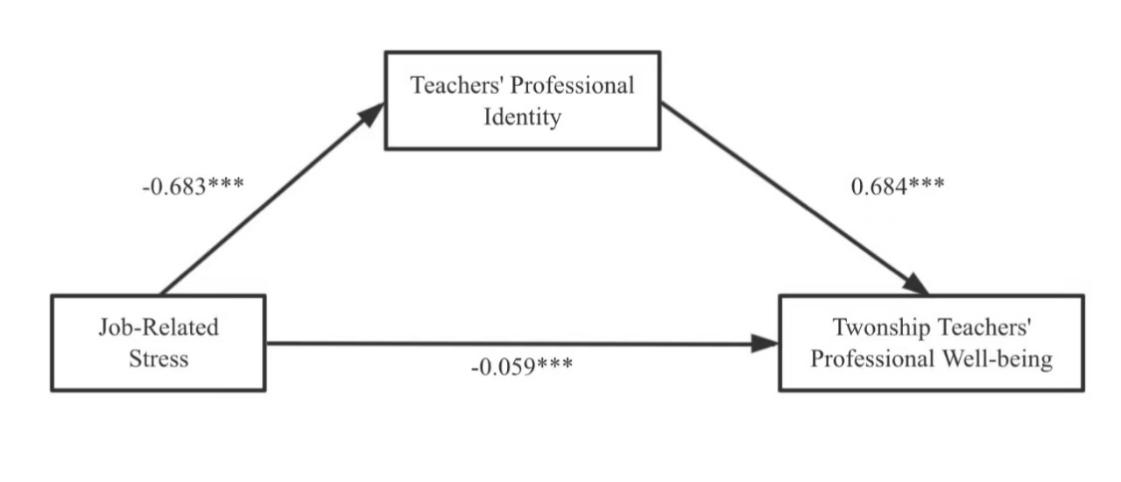


**Supplementary Figure 2.** Path models examining the mediation role of teachers’ professional identity between job-related stress and township teachers’ professional well-being. Standardized coefficients are presented. ****p* < 0.001.


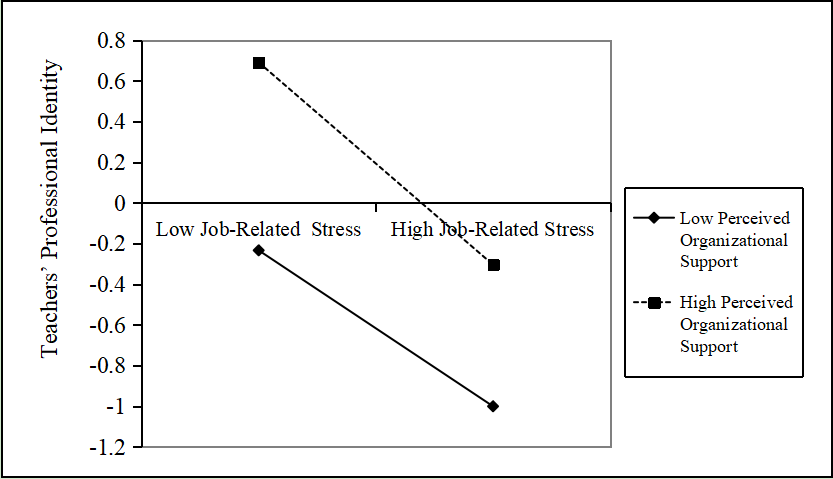


**Supplementary Figure 3.** Simple slope plot of the moderating effect of perceived organizational support

## Supplementary Tables

**Table 1**. Respondents’ Distribution

| Category | Items | Frequency | Percentage（%） |
| --- | --- | --- | --- |
| Gender | Male | 6191 | 25.50 |
|  | Female | 18085 | 74.50 |
| Age | 21-25 years | 1922 | 7.92 |
|  | 26-30 years | 3381 | 13.93 |
|  | 31-35 years | 3458 | 14.24 |
|  | 36-40 years | 3625 | 14.93 |
|  | 41-45 years | 4526 | 18.64 |
|  | 46-50 years | 3041 | 12.53 |
|  | 50-55 years | 3261 | 13.43 |
|  | 56-60 years | 1062 | 4.37 |
| Working Experience | 0-2 years | 3149 | 12.97 |
|  | 3-5 years | 2375 | 9.78 |
|  | 6-10 years | 2826 | 11.64 |
|  | 11-15 years | 2168 | 8.93 |
|  | 16-20 years | 2960 | 12.19 |
|  | 21-25 years | 4309 | 17.75 |
|  | 26-30 years | 2908 | 11.98 |
|  | 31-35 years | 2423 | 9.98 |
|  | 36-40 years | 1158 | 4.77 |
| Professional Title | Ungraded | 3313 | 13.65 |
|  | Third-level | 437 | 1.80 |
|  | Second-level | 6417 | 26.43 |
|  | First-level | 10034 | 41.33 |
|  | Senior | 4075 | 16.79 |
| Educational Background | College and below | 6457 | 26.60 |
|  | Undergraduate | 17337 | 71.42 |
|  | Postgraduate and PhD | 482 | 1.99 |
| Teaching Period | Senior High school | 1623 | 6.69 |
|  | Junior high school | 4660 | 19.20 |
|  | Primary school | 17993 | 74.12 |
| Types of School | Public school | 23828 | 98.15 |
|  | Private school | 448 | 1.85 |
| Boarding School | Yes | 9283 | 38.24 |
|  | No | 14993 | 61.76 |
